# Genetic Analysis, Transcriptome Analysis, and Candidate Major Genes Screening of Peduncle Length Trait in Brewing Sorghum [*Sorghum bicolor* (L.) Moench]

**DOI:** 10.3390/genes17040362

**Published:** 2026-03-24

**Authors:** Jinghua Li, Zunyan Hu, Zhiyong Hao, Bangsheng Sun, Zhouchen Ye, Guangdong Yang

**Affiliations:** 1 Keshan Branch of Heilongjiang Academy of Agricultural Sciences, Qiqihar 161005, China; lijinghua.168@163.com (J.L.); liping04230319@126.com (Z.H.); hzy19850712@126.com (Z.H.); sunbangsheng0451@163.com (B.S.); 2 College of Life Sciences, Agriculture and Forestry, Qiqihar University, Qiqihar 161006, China

**Keywords:** Sorghum, peduncle length, RNA-seq, DEGs, candidate major genes

## Abstract

Objectives: Peduncle length (PL) is a critical agronomic trait in sorghum [*Sorghum bicolor* (L.) Moench], influencing mechanical harvesting efficiency. Exploration of the PL genetic mechanism and the PL major genes of sorghum can provide a reference for breeding of sorghum suitable for mechanization and PL genetic research of other graminaceous crops. Methods: Here, we conducted genetic analysis, transcriptome analysis, and candidate major gene screening of PL using long-peduncle (KY133B) and short-peduncle (KY123B) parents, as well as their constructed F2 segregated populations. Results: Genetic analysis revealed that PL trait may be controlled by two major genes with additive-dominant effects, showing a heritability of 69.638%. At the early stage of sorghum peduncle elongation, the young panicle of the parents was sampled and performed transcriptome analysis. DEGs 3603 genes were obtained. With the short peduncle parent (F) as the control, 2204 upregulated genes and 1399 downregulated genes were expressed in the long peduncle parent (M). We compared the 1161 genes obtained by BSA-seq from the laboratory in the early stage with the DEGs obtained by RNA-seq, and obtained 148 co-localized genes. Through the high DEGs screening criteria (|Log_2_FC(M/F)| ≥ 5, *p* < 0.0001), we further identified 36 genes with highly significant expression differences between parents. Functional annotation identified four candidate major genes strongly associated with PL: *LOC8056900* (MIZU-KUSSEI 1), *LOC8065075* (ethylene-responsive transcription factor WIN1), *LOC8083493* (GDSL esterase/lipase), and *LOC8085367* (auxin-responsive protein IAA21). qPCR validated their expression trends, corroborating RNA-seq results. Conclusions: The comprehensive information presented here provides a reference for understanding the PL mechanism of sorghum and provides some important candidate major genes related to PL. This study laid the foundation for subsequent gene functional verification and mechanism analysis of sorghum peduncle length major genes.

## 1. Introduction

Sorghum [*Sorghum bicolor* (L.) Moench, 2*n* = 20] ranks as the fifth most extensively cultivated food crop globally, following maize, rice, wheat, and barley [1]. In the 1980s, the focus of sorghum breeding in China transitioned from high-yield varieties primarily intended for human consumption to specialized sorghum types suitable for brewing and animal feed. Brewing sorghum gradually became the main body of sorghum breeding and production, and the planting area accounted for about 85% of national sorghum production [2]. There is currently an urgent demand for the development of brewing sorghum varieties that possess high yield potential, adaptability to dense planting, strong resistance to environmental stresses, and compatibility with mechanized farming practices.

At present, the genetic and physiological mechanism of the traits of sorghum peduncle length is not clear. The sorghum peduncle, which emerges from the last node of the stem and connects the panicle and the stalk, functions as the sole pathway for transporting photosynthates produced by the stem and leaves to the panicle grains. Previous studies have demonstrated that peduncle length was negatively correlated with single-plant yield but positively associated with lodging resistance [3]. The length of peduncle has an important effect on the mechanized harvest of sorghum. Considering the critical role of the peduncle length, controlling peduncle length within an optimal range during breeding can enhance grain yield accumulation, improve lodging resistance, and facilitate mechanical harvesting.

Numerous studies have reported on quantitative trait loci (QTL) mapping for various agronomic traits in sorghum, including plant height, panicle length, post-flowering greening, root growth angle, grain traits, etc. [4,5,6,7,8,9,10]. However, research specifically targeting peduncle length remains limited, with most mapping methods relying on QTL mapping through genetic linkage maps. For example, Klein et al. [11] identified six QTLs associated with peduncle length using a sorghum genetic linkage map. Brown et al. [12] constructed a genetic map using recombinant inbred line (RIL) populations and located QTLs for 15 traits, including peduncle length. Zhai [13] detected a QTL on chromosome 6 related to peduncle length by constructing a genetic linkage map of sorghum, while Su [14] identified another QTL on chromosome 1 using an F6 generation population of recombinant inbred lines. More recently, Ding et al. [15] used a high-density genetic linkage map derived from an RIL population to identify 10 QTLs related to peduncle length, distributed across chromosomes 1, 3, 6, 7 and 8, respectively.

The QTL mapping based on a constructed genetic map has the characteristics of long construction time and high cost. Through the combined gene mapping technology of bulk segregant analysis (BSA-seq) and transcriptome analysis (RNA sequencing, RNA-seq), the number of candidate genes in the target region can be further narrowed, enabling rapid and efficient identification of key genes associated with specific traits. Joint gene mapping and candidate gene mining techniques using BSA-seq and RNA-seq have been successfully applied in species such as rice, soybean, and millet [16,17,18,19]. Gao et al. [18] found nine candidate genes related to plant height by using BSA and RNA-Seq combined analysis, and proposed a hypothetical mechanism for the formation of millet dwarfing, in which, metabolism and MAPK signaling play important roles in the formation of foxtail millet plant height based on the functional prediction of the candidate genes. Nan et al. [20] mapped the gene *BnaA5.AIB* by means of BSA-Seq and RNA-Seq, analyzing that this gene may be the key factor that links ABA to N signaling and a negative regulator of the N utilization efficiency. Geng et al. [21] found the gene *BnARF18* exhibited significantly differential expressions between parents by using BSA and RNA-Seq combined analysis, revealing that the MYC-motif, implicated in gene expression regulation, and the WUN-motif, associated with cell differentiation and proliferation control, likely serve as key regulatory motifs responsible for the differential expression levels of *BnARF18* between the two parental lines. However, the application to sorghum PL trait has not yet been reported. Therefore, in this study, we utilized the previously established BSA-seq to initially locate associated regions [2] and further employed RNA-seq to focus on differentially expressed genes within the target region. By integrating functional annotations, we identified candidate major genes regulating sorghum peduncle length. Consequently, this study is expected to provide novel insights into gene mapping for other quantitative traits in sorghum.

## 2. Materials and Methods

### 2.1. Plant Materials

**Figure 1:**
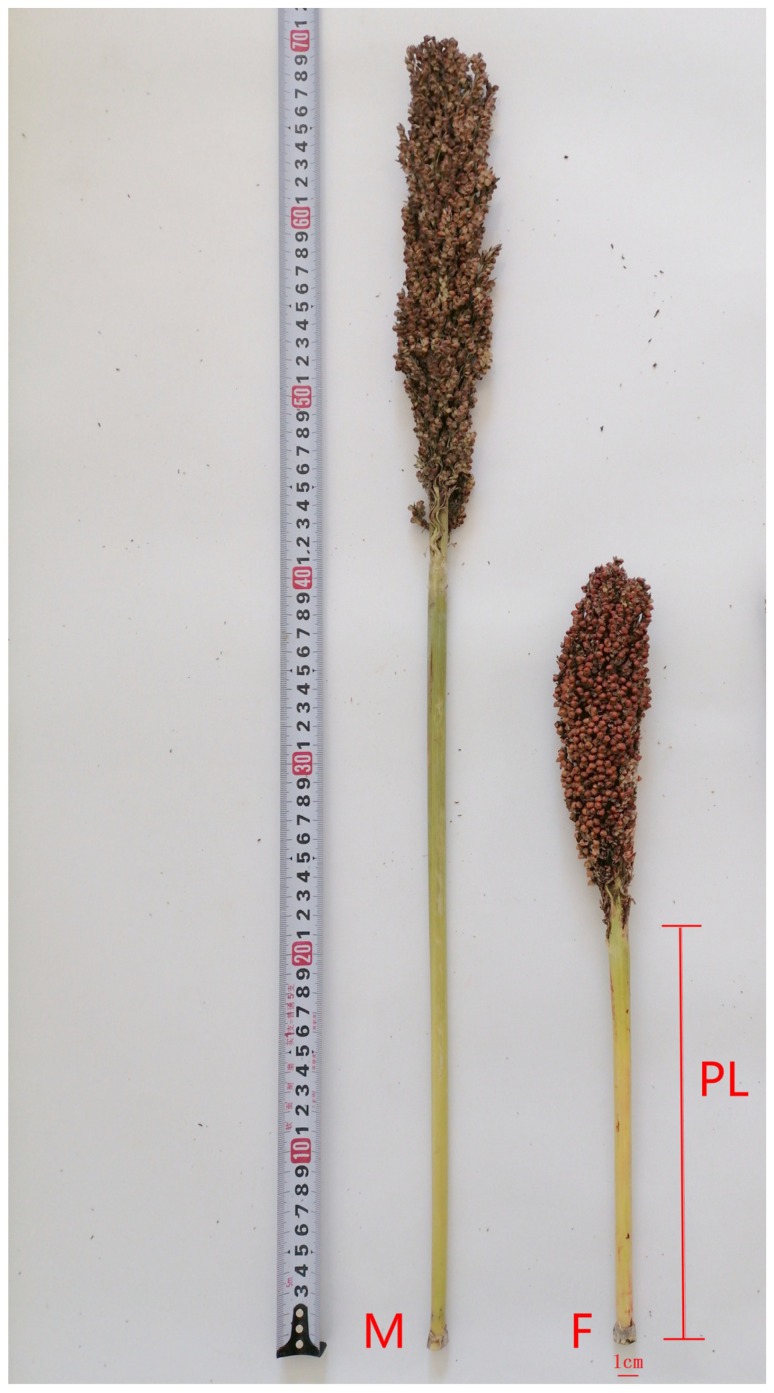
Long peduncle KY133B (M) and short peduncle KY123B (F).

Using long peduncle KY133B (maternal plant, M) and short peduncle KY123B (paternal plant, F) as parental lines (Figure 1), an F2 segregated population was constructed in this study. KY133B and KY123B were used for hybridization. After single panicle harvest, they were further planted and harvested (of the F2 generations). In May 2022, seeds from the KY133B, KY123B, and F2 groups were sown at the experimental planting base of Keshan Branch of Heilongjiang Academy of Agricultural Sciences (48°01′ N, 125°83′ E). A total of 339 F2 generation plants were harvested for further analysis. Plant spacing was 10 cm, row spacing was 65 cm, and row length was 2 m. The plants were grown in 1-row plots. Protective rows were established around the plants. Normal water and fertilizer management were conducted throughout the whole growth period.

### 2.2. Agronomic Traits Investigation and Genetic Analysis

For each plant in the F2 population, we measured several key traits, including plant height, peduncle length, panicle length, stem height, and primary branch length of the panicle. Specifically, plant height was defined as the vertical distance from the ground to the top of the main panicle; panicle length is measured as the distance from the base of the main panicle to its apex; peduncle length (PL) is defined as the distance from the last node of the stem and the base of the main panicle; stem height is defined as the vertical distance from the ground to the base of the main panicle; while the primary branch length of the panicle refers to the length of the primary branch located at the 2/3 position above the base of the entire main panicle.

Statistical analysis of sorghum peduncle length was performed using SPSS 16.0. Genetic analysis of the F2 isolated generation was conducted in accordance with the genetic model proposed by Wang et al. [22]. Data processing and analysis were carried out utilizing the R software package SEA v2.0 [23].

### 2.3. Transcriptome Sequencing and Differential Expression Genes (DEGs) Determination

Young panicles (collected in the booting stage of sorghum) from the parental plants were carefully collected for freezing in liquid nitrogen and stored in a −80 °C refrigerator for RNA extraction. Each parent included three biological replicates. Total RNA was extracted from the tissue using TRIzol^®^ Reagent (Invitrogen, Carlsbad, CA, USA) according to the manufacturer’s instructions. Then RNA quality was determined by 5300 Bioanalyser (Agilent, Santa Clara, CA, USA) and quantified using the ND-2000 (NanoDrop Technologies, Wilmington, DE, USA). The eukaryotic mRNA sequencing was performed on NovaSeq X Plus sequencing platform, capturing all mRNA transcribed by specific eukaryotic tissues or cells during a given time period. The sequencing libraries were prepared by Illumina^®^ Stranded mRNA Prep, Ligation (San Diego, CA, USA) method. Total RNA extraction and subsequent sequencing analysis were completed by Shanghai Majorbio Bio-Pharm Biotechnology Co., Ltd. (Shanghai, China).

To identify differentially expressed genes (DEGs) between two samples, we first quantified the expression level of each transcript using the transcripts per million reads (TPM) method with RSEM v1.3.3 (http://deweylab.github.io/RSEM/, accessed on 15 September 2023) [24]. Subsequently, differential expression analysis was conducted using the DESeq2 [25]. Genes with |log_2_FC| ≥ 1 and *P*adjust < 0.05 were identified as significantly differential.

### 2.4. Gene Ontology Analysis

DEGs were annotated with the corresponding terms in the Gene Ontology (GO) database [26]. The count of DEGs associated with each GO term was tallied to compile a list of genes with specific GO functions. Subsequently, a hypergeometric test was performed to identify significantly enriched GO (*P*adjust < 0.05) among the DEGs relative to the genomic background.

### 2.5. Pathway Enrichment Analysis

KEGG [27], a comprehensive public database for pathway information, was utilized to achieve this objective. In our study, pathway enrichment analysis revealed metabolic and signal transduction pathways that were significantly enriched among the DEGs compared to the whole-genome background. The adjusted *P*adjust were calculated using a false discovery rate (FDR) threshold of <0.05. Pathways satisfying this criterion were deemed significantly enriched in the DEGs.

### 2.6. Gene Function Annotation

The BLAST software v2.9.0 [28] was applied to compare with NR [29], GO [26], KEGG [27], EggNOG [30] and Uniprot [31] databases for in-depth annotation. The reference genome was Sorghum_bicolor_NCBIv3 version (https://www.ncbi.nlm.nih.gov/genome/108?genome_assembly_id=321335, accessed on 15 September 2023).

### 2.7. Validation of qPCR

Real-time PCR (quantitative real-time PCR, qPCR) was employed to validate the reliability of transcriptome results and the final candidate genes. Total young panicles RNA was extracted from the two parental samples. cDNA reverse transcription was performed using the HiScript Q RT SuperMix for qPCR (+gDNA wiper) (Vazyme, Nanjing, China) according to the manufacturer’s instructions. All qPCR analyses were conducted using ChamQ SYBR Color qPCR Master Mix (2X) on a fluorescence quantitative PCR instrument (ABI7300, Applied Biosystems, Carlsbad, CA, USA). Each parent included three biological replicates, and each biological replicate was analyzed with three technical replicates. The relative expression levels of target genes were calculated using the 2^−∆∆Ct^ method. The Actin gene (*LOC8062375*) was used as the internal reference gene. Primer design and synthesis were carried out by Shanghai Majorbio Bio-Pharm Biotechnology Co.; Ltd. (Shanghai, China) (Table 1).

## 3. Results

### 3.1. Genetic Analysis of PL Trait

**Figure 2:**
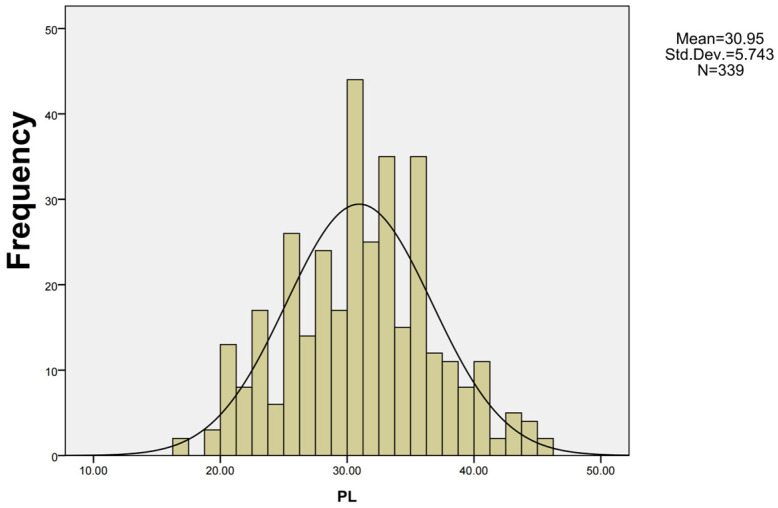
The PL frequency distribution in F2 population. Std.Dev. represents standard deviation. N represents sample size.

Using SPSS 16.0 software, we constructed the frequency distribution chart of the F2 segregating population consisting of 339 plants (Figure 2). The phenotype of the F2 segregating population followed a normal distributed, suggesting that peduncle length is a quantitative trait. To identify the most suitable genetic model for peduncle length, we evaluated several candidate models using goodness-of-fit tests, including U_1_^2^, U_2_^2^, U_3_^2^ (uniformity test), nW^2^ (Smirnov test), and Dn (Kolmogorov test). Based on Akaike’s Information Criterion (AIC) values, the optimal genetic model for peduncle length was determined to be the 2MG-AD (two pairs of additive-dominant major genes genetic model) (Table 2). This indicates that the peduncle length may be governed by two major genes, with additive effect values of 6.9866 and 1.9427, and dominant effect values of 0.3433 and 0.5033, respectively. The sum of the additive effect of the two main genes was significantly greater than the sum of their dominant effect (|da + db| > |ha + hb|), implying that the inheritance of peduncle length is primarily influenced by the additive effects of these two major genes. Furthermore, the heritability (h^2^) of peduncle length was estimated to be 69.638% (Table 3).

### 3.2. Correlation Analysis of PL Trait with Other Traits

Through correlation analysis among peduncle length, plant height, panicle length, stem height, and primary branch length of panicle, we found that peduncle length was highly significantly correlated with stem height, plant height, panicle length, and primary branch length of panicle. Notably, the correlations between peduncle length and both stem height and plant height were particularly strong, with correlation coefficients 0.83 and 0.81, respectively (Table 4). These results offer valuable insights into the regulatory mechanisms underlying the relationships among the length-related traits.

### 3.3. Transcriptome Analysis

#### 3.3.1. Quality Control of Transcript Sequencing Data

Young panicles were collected from paternal (F) and maternal (M) plants, with three biological replicates each, for eukaryotic reference transcriptome analysis. Using NovaSeq X Plus sequencing, a total of 299,811,088 raw reads were obtained from the parental samples. After quality control filtering, 295,507,060 clean reads were retained. The sequencing results demonstrated that the GC content of the data ranged from 52.2% to 52.76%, and the sequencing quality was high, with Q20 ≥ 94.63% and Q30 ≥ 91.57% (Table 5).

#### 3.3.2. Quality Assessment of RNA-Seq

**Figure 3:**
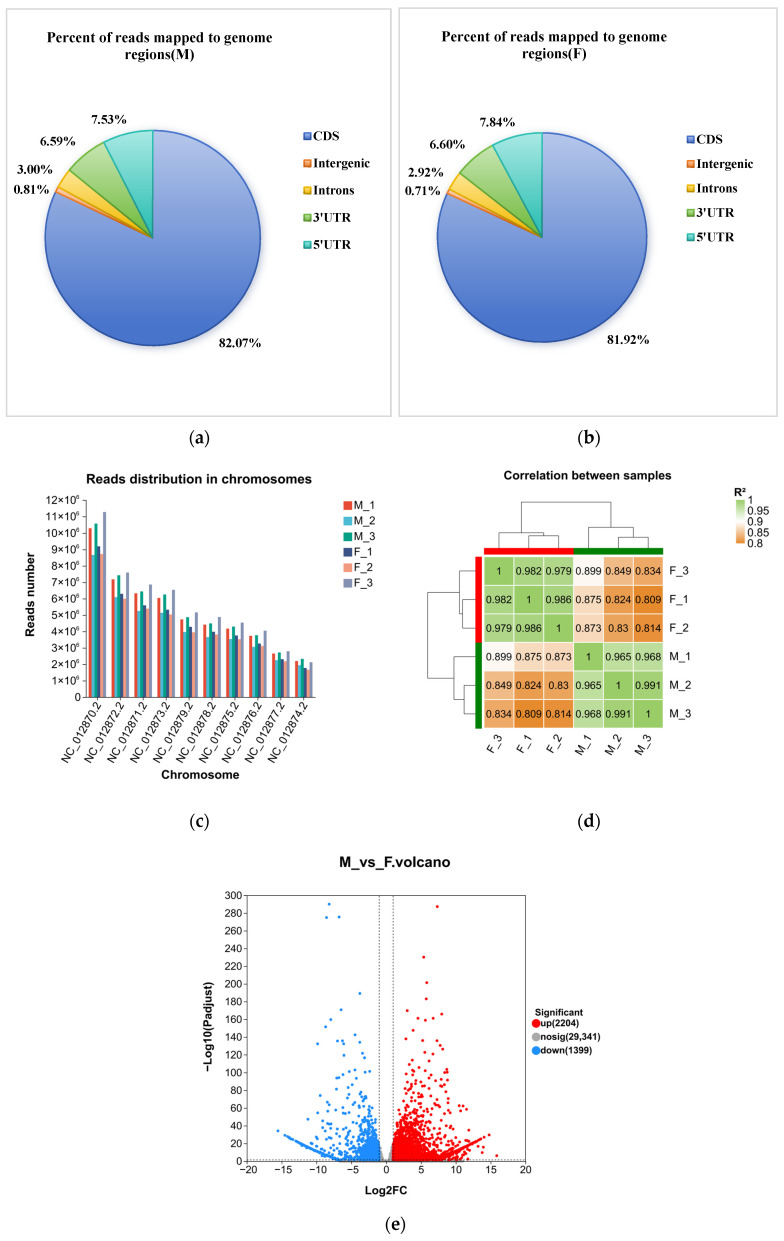
The results of reads mapping in two parents M (**a**) and F (**b**), reads distribution on chromosomes (**c**), the correlation analysis of six samples (**d**), and the differentially expressed genes between two parents (**e**).

The distribution of reads across the genome was calculated, and the mapping regions were categorized into CDS, intron, intergenic and UTR (5′ and 3′ untranslated regions). The majority of the mapped reads were concentrated in the CDS region of genes (Figure 3a,b). Analysis of reads distribution across different chromosomes revealed that the highest number of reads was observed on Ch.1 (NC_012870.2), while the lowest number of reads was found on Ch.5 (NC_012874.2) (Figure 3c). The biological repeatability and correlation of the samples were evaluated using the Pearson correlation coefficient, which confirmed the biological repeatability of the samples (Figure 3d).

#### 3.3.3. Identification of DEGs Related to PL

We quantified the expression levels of genes and transcripts using the RSEM software v1.3.3. Based on these quantitative results, we performed between-group differential gene analysis and identified genes with differential DEGs between the two parental lines. The differential expression analysis was conducted using DESeq2, with a screening threshold of |log_2_FC| ≥ 1 and *P*adjust < 0.05. A total of 3603 DEGs were identified, including 2204 up-regulated genes and 1399 down-regulated genes (Figure 3e).

#### 3.3.4. GO Classification and Enrichment Analysis

**Figure 4:**
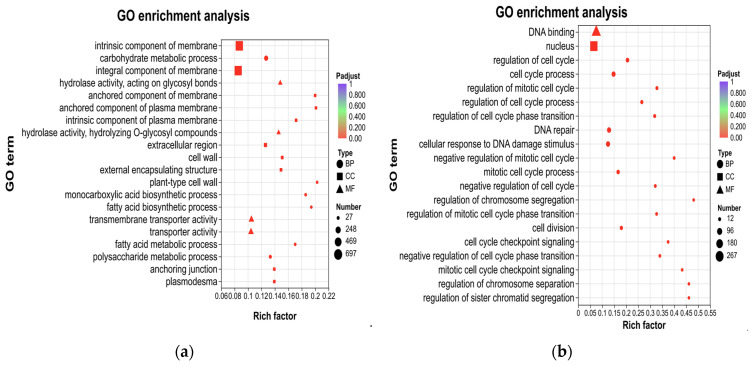
Gene Ontology (GO) enrichment analyses of DEGs obtained by RNA-seq. Top 20 GO terms enriched for upregulated DEGs in samples (**a**), and top 20 GO terms enriched for downregulated DEGs in samples (**b**). Among them, different colors represent the size of the *P*adjust, and the circular size represents the number of those enriched. MF represents molecular function, BP represents biological processes, and CC represents cellular components.

To obtain comprehensive functional information, GO enrichment analysis was conducted on both the upregulated and downregulated genes. The upregulated genes were significantly enriched in 76 terms, while the downregulated genes were significantly enriched in 182 terms (*P*adjust < 0.05). Among the top 20 significantly enriched functions, the upregulated DEGs were enriched in five terms related to biological processes (BP), four terms related to molecular functions (MF), and 11 terms related to cell components (CC). Functional descriptions with a high number of enriched genes included intrinsic component of membrane and integral component of membrane (Figure 4a). Among the top 20 significantly enriched functions, downregulated DEGs were mainly enriched in 18 terms related to biological processes (BP), one term related to molecular functions (MF), and one term related to cell components (CC). Functional descriptions with a large number of enriched genes also encompassed nucleus and DNA binding (Figure 4b). Based on these results, we hypothesized that the elongation of PL might be associated with the functional genes related to membrane components among the upregulated genes or with the DNA binding and nucleus-related functions among the downregulated genes.

#### 3.3.5. KEGG Classification and Enrichment Analysis

**Figure 5:**
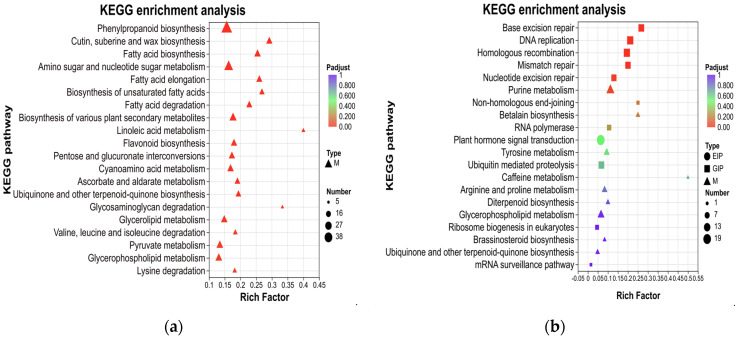
KEGG enrichment analyses of DEGs obtained by RNA-seq. Top 20 KEGG pathways enriched for upregulated DEGs in samples (**a**), and top 20 KEGG pathways enriched for downregulated DEGs in samples (**b**). Among them, different colors represent the size of the *P*adjust, and the circular size represents the number of those enriched. M represents metabolism, GIP represents genetic information processing, and EIP represents environmental information processing.

In order to functionally classify and assign pathways to genes associated with peduncle length, KEGG analysis was performed on all DEGs. The upregulated genes were significantly enriched in 22 pathways, whereas the downregulated DEGs were significantly enriched in five pathways (*P*adjust < 0.05). Among the top 20 significantly enriched functions, the upregulated DEGs were predominantly significantly enriched in metabolism pathways (M). Functional categories with a relatively higher number of enriched genes included phenylpropanoid biosynthesis, amino sugar and nucleotide sugar metabolism, as well as biosynthesis of various plant secondary metabolites (Figure 5a). For the top 20 significantly enriched functions, the downregulated DEGs were primarily enriched in genetic information processing pathways (GIP), including base excision repair, DNA replication, homologous recombination, mismatch repair, nucleotide excision repair, and purine metabolism (Figure 5b). Based on these findings, we hypothesized that the elongation of peduncle length might be associated with metabolism (M) or genetic information processing (GIP) pathways. Additionally, although not significantly enriched, a relatively larger number of downregulated genes were observed in the environmental information processing (EIP) pathway. The EIP pathway was the plant hormone signal transduction, indicating that the occurrence of PL in this study might also be related to hormone signal transduction.

### 3.4. Combined Analysis of RNA-Seq and BSA-Seq for Candidate Genes Prediction

**Figure 6:**
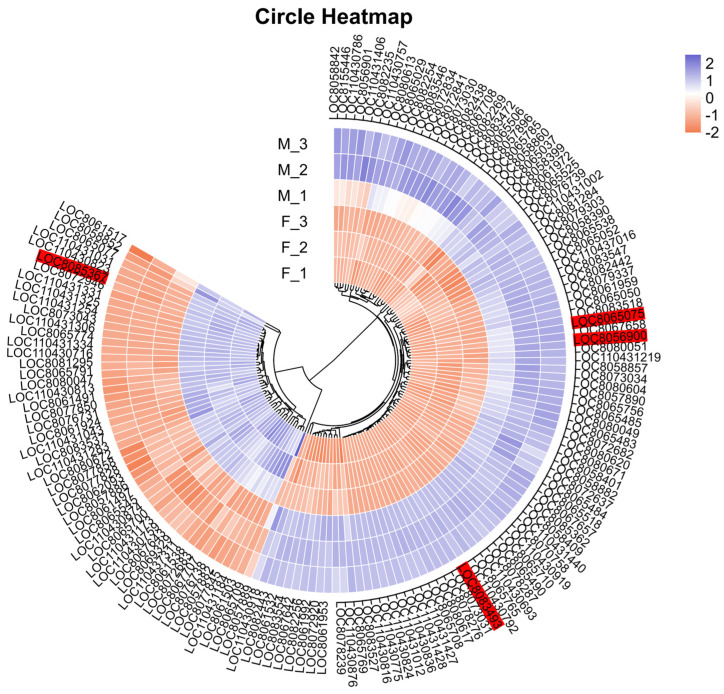
Heatmap generated from TPM values of the 148 co-localized genes in two parents. Heatmap of the expression of the 148 co-localized genes in two parents. Color indicates the expression size of the gene in the corresponding sample, red represents the higher expression of the gene in this sample, blue indicates the lower expression. For the specific trend, see the number annotation on the top right color bar. The cluster tree of the circle corresponds to the clustering of genes. The closer the branches of the two genes are, the closer the expression is. The four genes name numbered in labeled red are putative candidate major genes.

We compared the 1161 genes initially localized from BSA-seq from our laboratory’s earlier study [2] with the DEGs identified through RNA-seq. This comparison led to the identification of 148 co-localized genes, which are DEGs potentially associated with peduncle length traits. Among these, 20 genes were located on chromosome 7, while 128 genes were located on chromosome 10. Using the short peduncle parent (F) as the control, we observed that 99 genes were upregulated and 49 genes were downregulated in the long peduncle parent (M) (Figure 6).

**Figure 7:**
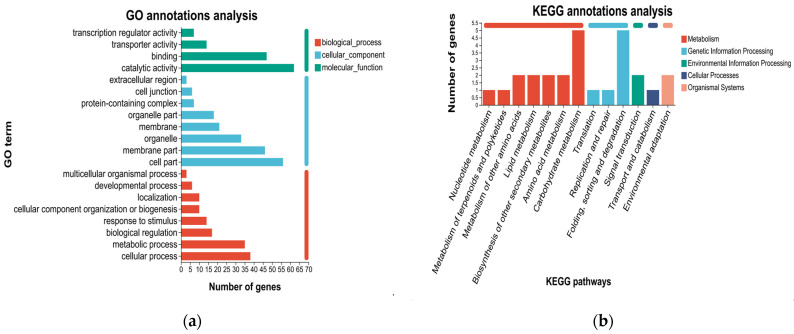
GO (**a**) and KEGG (**b**) annotations of 148 co-localization genes.

We performed GO and KEGG functional annotations for these 148 DEGs. Based on the GO function annotation results, a significant proportion of genes were enriched in the following categories: molecular functions (binding and catalytic activity), cellular components (organelle, membrane part, and cell part), and biological processes (metabolic process and cellular process) (Figure 7a). According to the KEGG annotation results, we identified 13 pathways distributed across four major categories. Specifically, seven pathways classified under metabolism (M), including nucleotide metabolism, terpenoid and polyketide metabolism, metabolism of other amino acids, lipid metabolism, biosynthesis of other secondary metabolites, amino acid metabolism, and carbohydrate metabolism. Three pathways categorized under genetic information processing (GIP), including translation, replication and repair, as well as folding-sorting-degradation. One pathway was linked to environmental information processing (EIP), specifically signal transduction. One pathway was categorized under cellular processes (CP), namely transport and catabolism. Lastly, one pathway belonged to organismal systems (OS), specifically environmental adaptation (Figure 7b).

We identified 36 genes with significant expression differences between parents by applying stricter screening criteria (|Log_2_ FC(M/F)| ≥ 5 and *p* < 0.0001) (Table 6). Function annotation of these 36 DEGs revealed four candidate major genes potentially strongly correlated with PL traits: *LOC8056900*, *LOC8065075*, *LOC8083493*, and *LOC8085367*. *LOC8056900* was located on chromosome 7, while the remaining three genes were located on chromosome 10. On chromosome 7, using the short peduncle parent (F) as the control, *LOC8056900* exhibited upregulated expression in the long peduncle parent (M). This gene encodes a MIZU-KUSSEI 1 (MIZ1) protein consisting of 268 amino acids and containing a domain of unknown function (DUF617). Its GO functional annotation is associated with hydrotropism. On chromosome 10, using the parent F as the control, *LOC8065075* and *LOC8083493* were upregulated in the parent M, whereas *LOC8085367* was downregulated. *LOC8083493* is a GDSL esterase/lipase gene with GO functional annotations including hydrolase activity and action on ester bonds. *LOC8065075* is an ethylene-responsive transcription factor WIN1 gene localized in the nucleus and containing an AP2/ERF domain. Its GO functional annotations include nucleus, DNA binding, transcription factor activity, and sequence-specific DNA binding. *LOC8085367* is an auxin-responsive protein IAA21 gene localized in the nucleus, with GO functional annotations including nucleus, regulation of transcription, DNA-templated, response to auxin, and auxin-activated signaling pathway. Its KEGG functional annotation is plant hormone signal transduction.

### 3.5. Analysis of RNA-Seq Data Reliability Using qPCR

**Figure 8:**
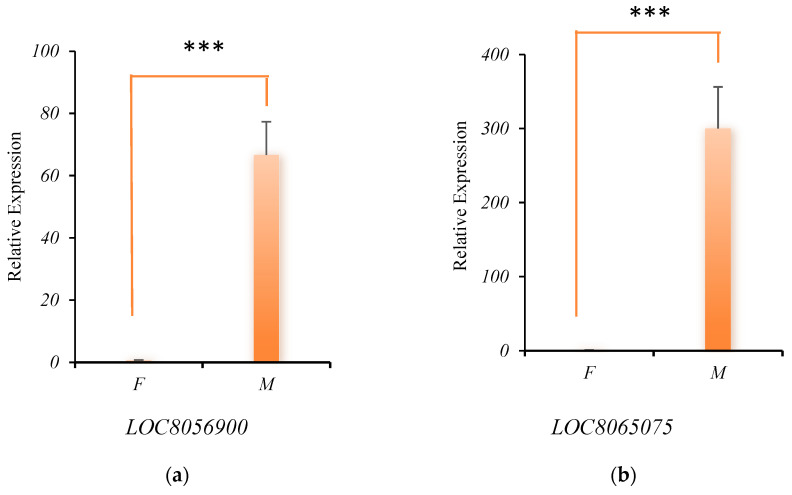

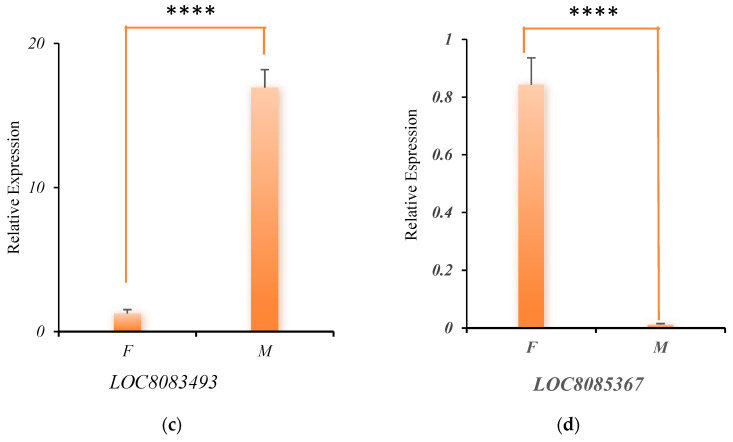
Analysis of expression of four candidate genes in parents by qPCR. The actin gene (*LOC8062375*) was used as an internal control. The transcription level of the short peduncle sample was set at 1.0. Asterisks indicate significant differences at various thresholds (***: *p* < 0.001; ****: *p* < 0.0001). Error bars represent the mean SD of three biological replicates. The (**a**–**d**) indicates the relative expression of four genes in different samples.

To verify the reliability of transcriptome data, we extracted RNA from young panicles of M and F parents and the expression of the four candidate genes was analyzed by qPCR. Using the parent F as the control, the expression of *LOC8056900*, *LOC8065075*, and *LOC8083493* was significantly upregulated in the parent M (Figure 8a–c), while the expression of *LOC8085367* was significantly downregulated (Figure 8d). These results demonstrated that the expression trends were consistent with the RNA-seq results (Table 5), thereby confirming the reliability of the transcriptome dataset.

## 4. Discussion

### 4.1. Genetic Analysis of PL in Sorghum

In contemporary genetic studies on PL of sorghum, the results remain inconsistent. Sun et al. [3] concluded that PL is a genetically complex trait. Their study revealed that, in addition to additive effect, PL exhibits 35.7% non-additive effect, which was susceptible to environmental conditions, and in addition to dominant effect, there was also non-allelic interaction in non-additive effect. Furthermore, Sun [32] demonstrated that PL is influenced by cytoplasmic effect. We found that the selected parental materials were different, resulting in different results [33,34]. Bai et al. [33] constructed an F2 population using grain sorghum as parents and identified that PL is controlled by an additive-dominant mixed genetic model involving one major gene, with a heritability rate of 61.58%. In another study, Bai et al. [34] used grain sorghum and Sudan grass as parents to construct an F2 population, revealing that PL was not a quantitative trait regulated by major genes but rather by microgenes. In our experiment, we selected brewing sorghum as the parental material to construct an F2 population, identifying the presence of two major genes with both additive and dominant effects, resulting in a heritability rate of 69.638%. The major gene has a large and stable effect, which is of great significance to the breeding efficiency and precision improvement of the PL trait in Sorghum.

### 4.2. Possible Regulatory Mechanism of PL-Related Genes in Sorghum

At present, no studies have specifically reported on genes associated with sorghum peduncle length, and the underlying regulation mechanism of this trait remains largely unclear. We searched related studies on PL in other graminaceous crops. Yan et al. [35] demonstrated that the deletion of two jasmonate (JA)-related genes resulted in significant elongation of maize peduncles, suggesting a potential link between PL and endogenous hormones. Some reports have indicated that the Rht 13 dwarf allele reduced plant height by altering wheat peduncle length, revealing a strong correlation or even causal relationship between plant height and PL trait [36,37]. In rice crops, the EUI (elongated uppermost internode) of recessive high rice mutants was found to exhibit rapid and enhanced internode elongation at the heading stage, especially in the uppermost internode [38]. Zhu et al. [39] discovered that the EUI mutants accumulated abnormally high levels of bioactive substances (gibberellins, GAs) in the uppermost internodes. Map-based cloning revealed that the EUI gene encodes an uncharacterized cytochrome P450 monooxygenase, CYP714D1. Liu et al. [40] concluded that the maize peduncle is a metamorphic stem with morphology and structure similar to the main stem. In this study, we observed a highly significant correlation between peduncle length, plant height, and stem height in sorghum. Therefore, we propose that the analyzing of the regulatory mechanism of PL should also involve studying genes influencing plant height of sorghum. So far, four genes regulating sorghum plant height have been reported: *Dw1*, *Dw2*, *Dw3*, and *Dw4* [41,42,43]. *Dw1* encodes a putative membrane protein with an unknown but highly conserved function in plants. *Dw2* encodes a protein kinase homologous to the AGCVIII protein kinase KIPK. *Dw3*, the first cloned dwarf gene in sorghum, encodes the auxin efflux transporter protein ABCB1. Although progress has been made regarding *Dw4*, the corresponding genes have not yet been cloned. Additionally, Girma et al. [44] identified the ethylene-responsive transcription factor RAP 2–7 as being significantly associated with sorghum plants, conferring delayed flowering time characteristics.

Based on the comprehensive analysis results of this study, we suggest that PL may be regulated by multiple genes, which is the result of the combined effects of hormone signals, transcriptional regulation, and environmental responses.

### 4.3. Functional Analysis of Candidate Major Genes

In this study, by searching co-localized genes based on the joint mapping of BSA-seq and RNA-seq, we finally inferred four possible candidate major genes. Among these, *LOC8056900*, *LOC8065075*, and *LOC8083493* were upregulated in long-peduncle parents, while *LOC8085367* was downregulated in long-peduncle parents.

*LOC8065075* is located on chromosome 10 and belongs to the *AP2/ERF* (*APETALA2*/Ethylene-responsive element binding factor) gene family. *AP2/ERF* transcription factors play important roles in plant growth, reproduction, and environmental interactions [45,46]. In rice, *OsEATB*, a member of the *AP2/ERF* gene family, has been reported to play a key role in regulating internode elongation. The crosstalk between ethylene and gibberellin mediated by *OsEATB* may underlie differences in internode elongation in rice. Studies have shown that overexpression of the *OsEATB* gene reduces rice plant height and panicle length at maturity, promotes branching potential in both tillers and spikelets, possibly via the regulation of both elongation and spikelet branching, thereby improving energy utilization and biomass yield [47]. Wondimu et al. [48] using genome-wide association analysis, identified an ethylene-responsive transcription factor *AP2/ERF* (*Sobic.003G324400*) as a strong candidate gene associated with sorghum plant height. *LOC8083493* belongs to the GDSL (Gly-Asp-Ser-Leu) lipase family, one of the subfamilies of lipase. Members of this family play a key role in regulating plant growth, development, and resistance to environmental stress; moreover, it is speculated that GDSL may participate in the regulation of germination, germ elongation, and flowering development by regulating cytokinin or gibberellin [49]. *LOC8085367* is a member of the Aux/IAA gene family, which belongs to the auxin early-response gene family. This gene family functions in plant tropic growth, lateral root development, organ development, apical dominance, and responses to biotic and abiotic stresses [50,51,52,53]. *LOC8056900* is located on chromosome 7 and encodes the MIZU-KUSSEI 1 (MIZ1) protein. It is hypothesized that MIZ1 has evolved to play an important role in adaptation to terrestrial life because hydrotropism could contribute to drought avoidance in higher plants [54]. Moriwaki et al. [55] suggest that MIZ1 may regulate water orientation by regulating endogenous auxin levels in roots. The MIZ1 in rice is considered to be involved in developmental processes and stress responses [56].

We suggest that the above four candidate major genes may play an important role in the hormone signal transduction and environmental response during the peduncle elongation process of sorghum. Currently, the functions of the aforementioned four candidate genes in sorghum have not been fully elucidated. Further function validation studies will be carried out in the future.

## 5. Conclusions

Peduncle length (PL) is a critical agronomic trait in sorghum, influencing mechanical harvesting efficiency. In this study, the long-peduncle (KY133B), short-peduncle (KY123B) parents, and their constructed F2 segregated populations, were utilized for genetic analysis for PL, revealed that PL trait may be controlled by two major genes with additive-dominant effects, showing a heritability of 69.638%. And by RNA-seq, DEGs 3603 genes were obtained. Based on the association regions identified by BSA-seq from the laboratory in the early stage, we obtained 148 co-localized genes, and found 36 genes with very significant expression differences between parents by high DEGs screening criteria (|Log_2_FC(M/F)| ≥ 5, *p* < 0.0001). Functional annotation identified four candidate major genes strongly associated with PL: *LOC8056900* (MIZU-KUSSEI 1), *LOC8065075* (ethylene-responsive transcription factor WIN1), *LOC8083493* (GDSL esterase/lipase), and *LOC8085367* (auxin-responsive protein IAA21). Through qPCR validation, the relative expression trends of these candidate genes were found to be consistent with the RNA-seq results. Regrettably, the correctness of the candidate major genes still requires further functional validation. Next, we will conduct validation studies such as overexpression and gene disruption.

The comprehensive information presented here provides a reference for understanding the PL mechanism of sorghum and provides some important candidate major genes related to PL, which lays the foundation for breeding of sorghum that is suitable for mechanization and PL research of other graminaceous crops.

## Figures and Tables

**Table 1:** Primer sequences used in qPCR.

Gene	Primer Sequence (5′-3′)
*LOC8056900*	F:AGGACTCTGAGGCGTTCTACAT R:TTGGCATCATGGCTGTTT
*LOC8065075*	F:ACCTGCGATGATGATGGA R:TCGGTGGTGTTTCAGATGAC
*LOC8083493*	F:CGACTACAACGCCAAGGTGC R:TGGAAGGGTTGGTGATGAGG
*LOC8085367*	F:TAGTGAATCTAATGGGAAATCG R:ATCCTGAGCCTCCTACAGC
*LOC8062375*	F:CCGACAACCTGATGAAGA R:TGAAGGATGGCTGGAATA

**Table 2:** Candidate model.

Traits	Candidate Model	Max Log Likelihood Value	Akaike’s Information Criterion
PL	0MG	−1073.079	2150.158
	1MG-AD	−1069.23	2146.46
	1MG-A	−1070.543	2147.085
	1MG-EAD	−1072.168	2152.337
	1MG-NCD	−1073.073	2154.146
	2MG-ADI	−1073.074	2166.148
	2MG-AD	−1065.205	2142.41
	2MG-A	−1073.075	2154.149
	2MG-EA	−1068.772	2143.544
	2MG-CD	−1073.08	2154.16
	2MG-EAD	−1073.08	2152.16

MG represents major gene. A represents additive effect. D represents dominant effect. N represents negative effect. I represents interaction. E represents equal additive. C represents complete effect.

**Table 3:** Genetic parameters analysis.

Trait	Model	d_a_	d_b_	h_a_	h_b_	i	j_ab_	j_ba_	l	h^2^	U_1_^2^	U_2_^2^	U_3_^2^	nW^2^	Dn
PL	0MG	_	_	_	_	_	_	_	_	_	0.0036	0.0099	0.0273	0.0666	0.0508
	1MG-AD	6.5813	_	0.876	_	_	_	_	_	50.3557	0	0	0.0019	0.0371	0.0327
	1MG-A	6.6465	_	_	_	_	_	_	_	57.8946	0.0386	0.0412	0.0026	0.0707	0.0497
	1MG-EAD	3.9002	_	_	_	_	_	_	_	28.3783	0.0069	0.0322	0.1561	0.0712	0.0463
	1MG-NCD	1.2071	_	_	_	_	_	_	_	3.6091	0.005	0.0141	0.0397	0.0679	0.0512
	2MG-ADI	1.4431	0.4761	−0.7245	−0.0006	0.0022	0.0028	0.7299	0.7219	3.8081	0.0037	0.0116	0.0386	0.0667	0.0507
	2MG-AD	6.9866	1.9427	0.3433	0.5033	_	_	_	_	69.638	0.0001	0.0003	0.0018	0.0316	0.0344
	2MG-A	0.9937	0.3047	_	_	_	_	_	_	4.4436	0.0055	0.0161	0.0483	0.0673	0.051
	2MG-EA	4.7633	_	_	_	_	_	_	_	69.3677	0.0003	0.0008	0.0305	0.0412	0.0405
	2MG-CD	1.0471	0.7472	_	_	_	_	_	_	4.0582	0.0026	0.0097	0.0382	0.0656	0.0503
	2MG-EAD	0.8979	_	_	_	_	_	_	_	3.9622	0.0027	0.0098	0.0382	0.0657	0.0504

d_a_ represents the additive effect of the major gene a. d_b_ represents the additive effect of the major gene b. h_a_ represents the dominant effect of the major gene a. h_b_ represents the dominant effect of the major gene b. i represents additive effect × additive effect of the 2 major genes. j_ab_ represents additive effect (a) × dominant effect (b). j_ba_ represents additive effect (b) × dominant effect (a). l represents dominant effect × dominant effect of the 2 major genes. h^2^ represents the heritability of the major gene. _ represents no value here.

**Table 4:** Correlation analysis of peduncle length with plant height, panicle length, stem height, and primary branch length of panicle.

Traits	Peduncle Length	Plant Height	Panicle Length	Stem Height	Primary Branch Length of Panicle
Peduncle length	1				
Plant height	0.81 **	1			
Panicle length	0.25 **	0.51 **	1		
Stem height	0.83 **	0.97 **	0.27 **	1	
Primary branch length of panicle	0.14 **	0.259 **	0.597 **	0.109 *	1

The asterisk indicates significant differences among different traits (*: *p* < 0.05; **: *p* < 0.01).

**Table 5:** Quality statistics of filtered reads.

Sample	Raw Reads	Raw Bases	Clean Reads	Clean Bases	Error Rate (%)	Q20 (%)	Q30 (%)	GC Content (%)
M_1	52,671,120	7,953,339,120	51,953,370	7,649,384,463	0.0307	94.63	91.62	52.51
M_2	44,286,172	6,687,211,972	43,685,832	6,435,363,597	0.0302	94.86	91.96	52.22
M_3	54,059,012	8,162,910,812	53,352,696	7,873,585,556	0.0302	94.87	91.96	52.76
F_1	46,871,512	7,077,598,312	46,238,394	6,830,035,389	0.0303	94.83	91.89	52.24
F_2	44,648,180	6,741,875,180	43,918,692	6,437,726,096	0.0308	94.64	91.57	52.2
F_3	57,275,092	8,648,538,892	56,358,076	8,175,900,225	0.0296	95.15	92.39	52.25

Q20 (%) represents percentage of bases with sequencing quality above 99% of the total base count. Q30 (%) represents percentage of bases with sequencing quality above 99.9% of the total base count. GC content (%) represents the percentage of total G + C base count in quality control data relative to total base count.

**Table 6:** Candidate gene information statistics.

Entrez Gene ID	Chromosome	Gene Description	TPM (M)	TPM (F)	Log_2_ FC(M/F)	Regulate
*LOC8083527*	7	transcript variant X1	25.75	0.00	12.39	up
*LOC8083518*	7	probable potassium transporter 4, transcript variant X1	11.66	0.30	5.29	up
*LOC8080617*	7	-	17.04	0.00	10.90	up
*LOC8056900*	7	protein MIZU-KUSSEI 1	6.11	0.07	6.59	up
*LOC8085367*	10	auxin-responsive protein IAA21, transcript variant X1	2.02	139.13	−6.06	down
*LOC8083493*	10	GDSL esterase/lipase At4g26790	18.60	0.21	5.87	up
*LOC8082235*	10	acyl transferase 10	6.49	0.15	5.41	up
*LOC8080049*	10	26.2 kDa heat shock protein, mitochondrial	45.91	1.25	5.17	up
*LOC8079337*	10	cyanidin 3-O-rutinoside 5-O-glucosyltransferase	4.07	0.00	9.18	up
*LOC8078276*	10	-	56.73	0.00	12.33	up
*LOC8073043*	10	transcript variant X1	0.00	300.51	−15.53	down
*LOC8073031*	10	-	1.35	0.00	8.01	up
*LOC8073030*	10	transcript variant X1	3.13	0.01	8.10	up
*LOC8072834*	10	aspartyl protease family protein At5g10770	2.86	0.00	8.62	up
*LOC8065774*	10	transcript variant X1	0.00	12.68	−10.88	down
*LOC8065708*	10	transcript variant X12	50.46	0.76	5.55	up
*LOC8065485*	10	26.2 kDa heat shock protein, mitochondrial	454.28	10.51	5.43	up
*LOC8065075*	10	ethylene-responsive transcription factor WIN1	27.09	0.04	9.44	up
*LOC8065050*	10	crocetin glucosyltransferase, chloroplastic	4.71	0.00	9.33	up
*LOC8065029*	10	beta-galactosidase 9	61.97	1.64	5.23	up
*LOC8061924*	10	-	0.00	0.86	−8.06	down
*LOC8057896*	10	-	7.79	0.01	9.19	up
*LOC110431440*	10	-	18.47	0.16	8.63	up
*LOC110431428*	10	transcript variant X2	18.96	0.02	9.99	up
*LOC110431427*	10	transcript variant X1	10.73	0.00	10.53	up
*LOC110431334*	10	transcript variant X1	0.02	16.49	−9.20	down
*LOC110431331*	10	transcript variant X1	0.12	34.05	−8.17	down
*LOC110431325*	10	transcript variant X3	0.07	16.85	−7.01	down
*LOC110431306*	10	transcript variant X1	0.00	20.44	−12.22	down
*LOC110431012*	10	-	107.05	0.00	12.23	up
*LOC110430836*	10	transcript variant X1	47.90	0.01	11.15	up
*LOC110430824*	10	transcript variant X1	17.12	0.00	11.23	up
*LOC110430816*	10	-	3.22	0.00	9.05	up
*LOC110430775*	10	-	39.15	0.00	11.40	up
*LOC110430716*	10	transcript variant X1	1.00	201.02	−8.18	down
*LOC110430693*	10	transcript variant X2	112.47	0.43	8.36	up

TPM (M) represents transcripts per million from M sample. TPM (F) represents transcripts per million from F sample. FC represents fold change. The areas labeled in red are information statistics of the putative candidate major gene.

## Data Availability

The original contributions presented in this study are included in the article. Further inquiries can be directed to the corresponding authors.
